# Operationszeitpunkt bei COVID-19-positiven Patienten: Wann sollen wir operieren?

**DOI:** 10.1007/s00053-021-00558-w

**Published:** 2021-08-26

**Authors:** Irmgard E. Kronberger

**Affiliations:** Center of Operative Medicine, Department of Visceral, Transplant and Thoracic Surgery, Medical University Innsbruck, Anichstr. 35, 6020 Innsbruck, Österreich

## Originalpublikation

COVIDSurg Collaborative, GlobalSurg Collaborative (2021) Timing of surgery following SARS-CoV‑2 infection: an international prospective cohort study. Anaesthesia. 76(6):748–758. 10.1111/anae.15458. (Epub 2021 Mar 9. PMID: 33690889, PMCID: PMC8206995).

## Einleitung.

Bei anhaltender Pandemie und kumulativ steigender Anzahl von Menschen mit SARS-CoV-2-Diagnose wächst die Anzahl von Patienten, die während oder nach einer solchen Infektion einer Operation bedürfen oder eine planen möchten. Patienten nach respiratorischen Infekten, insbesondere nach einer SARS-CoV-2-Erkrankung, haben ein erhöhtes Sterblichkeitsrisiko und vermehrt postoperative pulmonale Komplikationen. Das Ziel dieser Studie war es, den optimalen Zeitpunkt für eine Operation nach einer SARS-CoV-2-Infektion zu bestimmen.

## Methodik.

In der internationalen, multizentrischen und prospektiven Kohortenstudie wurden Patienten eingeschlossen, die sich einer Operation in Allgemeinnarkose unterzogen. Es wurden im Oktober 2020 wochenweise nach Disziplin definierte, chirurgische Patienten eingeschlossen. Jene, die sich postoperativ mit SARS-CoV‑2 infizierten, wurden von der Studie ausgeschlossen. Primäres Studienziel war das postoperative 30-Tage-Überleben.

## Ergebnisse.

Die Zeit von der SARS-CoV-2-Diagnose bis zur Operation betrug bei 1144 (0,8 %) Patienten 0–2 Wochen, bei 461 (0,3 %) 3–4 Wochen, bei 327 (0,2 %) 5–6 Wochen und bei 1205 (0,9 %) 7 oder mehr Wochen. 137.590 (97,8 %) hatten keine SARS-CoV-2-Infektion; deren bereinigte 30-Tage-Sterblichkeit betrug 1,5 %. Eine erhöhte 30-Tage-Sterblichkeit zeigte sich bei Patienten, deren SARS-CoV-2-Infektion 0–2 Wochen (4,0 %), 3–4 Wochen (4,0 %) und 5–6 Wochen (3,6 %) zurücklag, nicht aber bei Patienten, die 7–8 Wochen nach der SARS-CoV-2-Diagnose operiert wurden (1,5 %). Bei einer Verzögerung des Operationszeitpunktes von 7 Wochen oder mehr hatten Patienten mit anhaltenden COVID-19-Symptomen eine höhere Sterblichkeit (6,0 %) als jene mit abgeklungenen Symptomen (2,4 %) oder asymptomatischen Patienten (1,3 %).

## Schlussfolgerung.

Patienten, die bis zu 6 Wochen nach Diagnose der SARS-CoV-2-Infektion operiert wurden, weisen ein erhöhtes postoperatives Sterberisiko und pulmonales Komplikationsrisiko auf. Dies gilt auch für Patienten mit anhaltenden Krankheitszeichen der Infektion zum Zeitpunkt der Operation.

## Kommentar

Obwohl bereits länger bekannt ist, dass eine perioperative Infektion mit SARS-CoV‑2 die postoperative 30-Tage-Mortalität und das Risiko für pulmonale Komplikationen erhöht [1] und internationale Richtlinien empfehlen, die Operation bei diesen Patienten zu verschieben, gibt es nur wenige Erkenntnisse über die optimale Dauer einer solchen Operationsverschiebung. Dieser Frage nahmen sich die GlobalSurg/CovidSurg Kollaboration an. Mehr als 25.000 Mitglieder der COVIDSurg Kollaboration sammelten Daten von 140.727 Patienten in 1674 Krankenhäusern und 116 Ländern. Das Gelingen von einem Unterfangen dieser Größenordnung hängt einerseits an der ausdauernden Arbeit der Studienzentrale an der Universität Birmingham, vor allem aber auch von der eifrigen Nutzung aller sozialen Netzdienste und der Mitarbeit von vielen Studenten und jungen Ärzten in den von je einem Oberarzt angeführten Teams der verschiedensten chirurgischen Disziplinen ab. Ethikanträge und Problemlösungen wurden intern rasch bekanntgegeben, länderspezifische Gegebenheiten diskutiert, Übersetzungen gemeinsam gemacht und die Dokumente oder Hilfestellungen online verfügbar gemacht. Die Globalität dieser Zusammenarbeit zeigt sich nicht zuletzt in der Teilnahme der sonst stark unterrepräsentierten Low-income-Länder – weniger als 10 % der Forschung über COVID-19 findet bisher in Low-income-Ländern statt.

Der primäre Endpunkt war die postoperative 30-Tage-Mortalität, sekundärer Endpunkt die Inzidenz von postoperativen pulmonalen Komplikationen (Pneumonie, akutes Atemnotsyndrom [ARDS] und/oder unerwartete postoperative Beatmung) nach 30 Tagen. Angesichts der hohen Zahl an teilnehmenden Patienten in dieser Studie und vor allem nach Bereinigung der Sterblichkeitsdaten (Alter, Geschlecht, ASA, Revised Cardiac Risk Index, respiratorische Komorbidität, Grad und Dringlichkeit der Operation, Ländereinkommen und Zeitpunkt der Operation nach SARS-CoV-2-Diagnose) kann die Studie eine solide Antwort auf die Fragestellung geben. Die Werte sind in den Subgruppenanalysen konsistent, der Datensatz weist eine hohe Vollständigkeit auf. Bei den pulmonalen Komplikationen zeigt sich die Angleichung der Risiken an jene eines Patienten ohne präoperativ stattgehabter Sars-Cov-2-Infektion ebenfalls ab der 7. Woche. Die Autoren(gruppe) der CovidSurg Kollaboration folgert daraus die Empfehlung zur Verschiebung einer Operation um mindestens 7 Wochen nach Infektion, bei anhaltenden Symptomen einer SARS-CoV-2-Infektion auch darüber hinaus.

Angesichts der dringenden Fragestellung nach der Dauer einer Verschiebung einer Operation für Millionen von Menschen seit Beginn dieser Pandemie wurden die Studienergebnisse sehr rasch aufgearbeitet und bereits am 10. März 2021 im „Anaesthesia“ online first und für jedermann verfügbar gemacht. Viele nationale und internationale Gremien haben diese Studienergebnisse daraufhin in ihre Empfehlungen aufgenommen.

Nicht nur die Kollaboration empfiehlt eine gemeinsame Entscheidungsfindung (interdisziplinär und mit dem Patienten) bezüglich des Zeitpunkts einer Operation nach einer SARS-CoV-2-Infektion. Jedenfalls muss im Entscheidungsprozess der Schweregrad der Infektion und eventuell anhaltende Symptome von COVID-19, die Komorbiditäten und der funktionelle Status des Patienten, die klinische Priorität und das Risiko eines Fortschreitens der Erkrankung sowie die Komplexität der Operation berücksichtigt werden [2]. Eine elektive Operation sollte nach den aktuell verfügbaren Ergebnissen nicht innerhalb von 7 Wochen nach der Diagnose einer SARS-CoV-2-Infektion (bzw. eines positiven Tests) geplant werden, es sei denn, die Risiken einer Verschiebung der Operation überwiegen das Risiko einer postoperativen Morbidität oder Mortalität im Zusammenhang mit COVID-19.

Die große CovidSurg-Studiengruppe zeigt auf, wie dringende Fragestellungen während einer Pandemie anhand einer globalen Kohortenstudie angegangen werden können und dass Co-Autorenschaften an alle (!) mitwirkenden Forscher ergehen können. Letzteres lässt eine neue Art der Handhabung von „Gruppenarbeiten“ in der Welt der Forschung und Autorenschaften erahnen und lässt die Motivation angehender Ärzte – auch in kleineren Krankenhäusern oder Arztpraxen –, in der Forschung mitzuwirken, steigen. Vor allem ist sie ein gut funktionierendes und diskussionsfreudiges chirurgisches Forschungsnetzwerk, das alle chirurgischen Disziplinen und auch anästhesiologische Kollegen einschließt und nicht nur wissenschaftlich, sondern auch für die Rekrutierung junger Kollegen von Bedeutung ist.

## References

[1] Collaborative CO (2020). Mortality and pulmonary complications in patients undergoing surgery with perioperative SARS-CoV-2 infection: an international cohort study. Lancet.

[2] El-Boghdadly K, Cook TM, Goodacre T (2021). SARS-CoV-2 infection, COVID-19 and timing of elective surgery: a multidisciplinary consensus statement on behalf of the Association of Anaesthetists, the Centre for Peri-operative Care, the Federation of Surgical Specialty Associations, the Royal College of Anaesthetists and the Royal College of Surgeons of England. Anaesthesia.

